# Effect of biological treatment used before harvesting and storage methods on the quality, health and microbial characteristics of unripe hazelnut in the husk (*Corylus avellana* L.)

**DOI:** 10.7717/peerj.12760

**Published:** 2022-01-28

**Authors:** Bogumił Markuszewski, Anna Adriana Bieniek, Urszula Wachowska, Arkadiusz Bieniek, Izabela Krzymińska

**Affiliations:** 1 Department of Agroecosystems and Horticulture, University of Warmia and Mazury in Olsztyn, Olsztyn, Warmia and Mazury, Poland; 2 Department of Entomology, Phytopathology and Molecular Diagnostics, University of Warmia and Mazury in Olsztyn, Olsztyn, Warmia and Mazury, Poland; 3 Department of Soil Science and Microbiology, University of Warmia and Mazury in Olsztyn, Olsztyn, Warmia and Mazury, Poland; 4 Institute of Microbiological Technologies in Turek, Turek, Poland

**Keywords:** Fresh hazelnut, *Arthrobacter*, *Pantoea*, Probiotics, *Pythium oligandrum*, *Cladosporium*

## Abstract

**Background:**

The hazelnut (*Corylus avellana*) is still one of the most profitable nut crop species. In recent years, however, there has been growing interest in this species in the form of “fresh nuts” that are picked before falling out of the fruit cover. The aim of this study was to evaluate the effect of storage conditions for hazelnuts protected with biological preparations on selected morphological features of the fruits, their health status and the count of bacteria and fungi colonizing the fruits.

**Results:**

The hazelnuts harvested from the trees protected with a preparation containing *Pythium oligandrum* and stored for 2 months under the controlled atmosphere conditions and in Xtend® bags (MAP) had the greatest weight and the highest percentage of the kernel. After 3 months of storage, the hazelnuts had reduced commercial value. Only a few hazelnuts displayed symptoms of infectious diseases caused by species of *Botrytis* and *Monilia*. The protection applied before the hazelnut harvesting contributed to a multiple increase in the bacterial and yeasts count on the husks and shells of the hazelnuts stored for 3 months. The bacterial count on the nuts stored under the controlled atmosphere (CA, 3%O_2_:3%CO_2_, a temperature of 0–1 °C, humidity of 85–95%) and under the controlled atmosphere conditions and in Xtend® bags (MAP) increased significantly. An analysis of the ITS region sequence revealed the presence of bacteria *Arthrobacter luteolus* and *Pantoea agglomerans*. A Koch test proved that both non-pathogenic bacteria and pathogenic fungi can cause the browning of the *C. avellana* leaf under conditions of high humidity. The application of a controlled atmosphere is recommended for a short-term storage of hazelnuts in the husk.

**Conclusion:**

This research showed that 2 months’ storage of hazelnuts under controlled atmosphere conditions and Xtend® bags (MAP) prevented a reduction in the weight of hazelnuts in the husk, without the husk, and of the kernel and prevented the nut separation from the husk. In general, the application of biopreparations for the protection of the hazelnut had a positive effect on the kernel weight and size.

## Introduction

In Europe, most hazelnuts (*Corylus avellana* L.) are cultivated in Turkey and Italy. In Poland, the area under hazelnut cultivation amounts to approx. 4000 ha. The consumption of fresh hazelnuts immediately after the harvesting is low; most of the hazelnut yield is intended after drying for the food industry (Zdyb, 2010; Ghirardello et al., 2013).

The hazelnut is a fruit which is usually consumed after it has reached harvest maturity and is separated from the husk and dried. After the proper partial drying of nuts following the harvesting to a 5% moisture content, they can be stored with a minimum loss of quality at 0–2 °C and 60–70% relative humidity for as long as 2 years (Ebrahem, Richardson & Tetley, 1994; Leuty et al., 2012; Ghirardello et al., 2013). Storage of hazelnuts at temperatures above 10 °C and humidity above 70% contributes to significant losses after severe infection by fungi, some of which can produce mycotoxins that can be dangerous to human health (Şimşek, Arici & Demir, 2002; Campbell, Molyneux & Schatzki, 2003; Navarro, 2006). Controlled and modified atmospheric conditions ensure the maintenance of high ripe nut quality during storage (Keme et al., 1980; San Martin et al., 2001; Mencarelli et al., 2008; Massantini & Contini, 2009; Ghirardello et al., 2013).

Hazelnuts in the husk that are harvested before they reach physiological maturity are becoming increasingly popular and available commercially. In such a form, they are offered usually for sale from August to September. Hazelnuts in the husk have higher water content, and their weight may be greater by up to 41% (Ciemniewska-Żytkiewicz et al., 2015; Markuszewski et al., 2017). Their kernels have higher contents of polyphenols and certain fatty acids. Moreover, they possess better gustatory properties (Ebrahem, Richardson & Tetley, 1994; Farinelli et al., 2001; Ciemniewska-Żytkiewicz et al., 2015). Due to their incomplete ripeness and a higher moisture content, the fresh in the husk hazelnuts have a shorter storage period than ripe hazelnuts, yet under controlled and modified conditions they can be stored for up to several months. The storage of fresh hazelnuts in Xtend® bags reduces oxygen concentration and increases carbon dioxide concentration as a result of their respiration. At the same time, the relative humidity increases, and the controlled diffusivity of the Xtend® bag material allows the relative humidity to be maintained at a level of 90–95%, which is very beneficial for the storage of fresh products (Aharoni et al., 2008). As result of the long period of storage of fresh hazelnuts in Xtend® bags, the high humidity contributes to severe infection of hazelnuts in the husk by storage pathogens (Markuszewski & Kopytowski, 2015).

The storability of fresh hazelnuts is also largely determined by the cultivar (Moscetti et al., 2012; Markuszewski & Kopytowski, 2015). Nuts kept under room temperature (24 ± 2 °C) conditions may rapidly lose their kernel moisture content, which contributes to a change in their colour and progressive rancidity as well as a reduction in the antioxidant activity (Ballisteri, Arena & Fallico, 2009; Christopoulos & Tsantili, 2012). The duration of fresh hazelnut storage period may also depend on the harvest date: the later the hazelnuts are harvested, the less hydrated the husk (Zdyb, 2010).

In Poland, few dangerous hazel diseases have been reported as occurring during the growth period: moniliasis (*Monilinia coryli* Schellenb. Honey) (Broniarek-Niemiec, 2020), grey mould (*Botrytis cinerea*), powdery mildew (*Phyllactinia corylea*) and dwining of hazels’ buds and hazels’ young stem shoots caused by the pathogen *Cryptosporiopsis coryli* (Machowicz-Stefaniak & Zalewska, 2002). In addition to infections by *Botrytis cinerea* and *Monilinia coryli* during the storage period, nuts are also get infected and decay by bacteria of the genus *Erwinia* (Król, Machowicz-Stefaniak & Zalewska, 2004). In 2007, and for the first time in Poland the bacterial blight of hazelnut caused by *Xanthomonas arboricola* pv. *corylina* (Pulawska et al., 2010) was recorded. However, because of the very low acreage of hazelnut crop in Poland phytopathological literature is very limited. In the USA hazelnut trees are being affected by pathogens leading to trunk cankers (*Diplodia mutilla*, *Dothiorella omnivore*, *Valsa* cf. *eucalypti* and *Diaporthe eres*) (Wiman et al., 2019) and endemically the encountered *Anisogramma anomola* pathogen leading to eastern filbert blight (Muehlbauer et al., 2018). In Italy cystospore canker is caused by at least three agressive pathogenic fungi *Anthostoma decipiens*, *Diaporthella cryptica* and *Diplodia seriata* (Linaldeddu et al., 2016), and the culprit of bacterial canker is *Pseudomonas syringae* pv. *corylina* bacterium (Lamichhane et al., 2013; Scortichini et al. 2016). During storage nuts can also show symptoms of dwining and tissue decay caused by toxic species *Alternaria alternata*, *Fusarium sporotrichiodes* (Duran et al., 2020) and *Aspergillus flavus* (Ozay et al., 2008). In contradiction to other countries, in Poland not much research that diagnose the reasons of dwining trees of *C. avellana* during growing period and the decay of nuts during storage period were done until now. Considering the increasing demand by consumers for nuts the knowledge about pathogens decaying nuts and ways of storage and protection from pathogens needs to be completed and extended. The available literature on the effect of biological control on the health status of horticultural plants demonstrates that they limit the occurrence of pathogens during the growing period; however, their effect can be altered by meteorological conditions (Mohamed et al., 2007; Rekanovic et al., 2007; Wang, Lou & Xu, 2011; Boček et al., 2012). Only a few studies concern the quality of stored fruits after the application of biological preparations (Bryk & Rutkowski, 2012).

The aim of this study was to evaluate the effect of storage conditions for hazelnuts protected with biological preparations on selected developmental stages of the fruits, the health status of the fruit, and the count of bacteria and fungi colonising these fruits.

## Materials and Methods

### Origin of hazelnuts in storage

The study used hazelnuts in the husk of the ‘Kataloński’ cultivar originating from a production orchard in Tuszewo (53°47′02″N, 19°78′16″E), located in north-eastern Poland where the average annual precipitation amounts to 473.0 mm and the average temperatures during the growing season are 8.3 °C in April, 12.7 °C in May, 15.3 °C in June, 19.5 °C in July, 19.6 °C in August, and 14.4 °C in September.

### Protective treatments were applied during the growing season

The hazelnuts fruit were harvested manually at the beginning of September, 2–3 weeks before they reached physiological maturity from trees of 8–years old (in 2017 and 2018). All hazelnuts used at harvest were in the husk. The trees had been planted on a sandy soil at a spacing of 5 × 2 m and prepared as low-headed trees in a spindle form. The soil in the tree rows was mulched with straw, and grass was maintained between the rows. The trees from which the hazelnuts were picked for the study grew in three rows of 60 trees each. The control in this experiment were considered the trees sprayed four times with the fungicides listed in Table 1. Additional treatments using microorganisms were introduced in the initial (BBCH 69 and 71) and final (BBCH 85, 88 and 90, Meier, 2003) phases of this trial. The probiotics were a mixture of living microorganisms SCD ProBio Plus® with lactic acid and photosynthetic bacteria, fermenting fungi and yeasts (EmFarma Plus™ and Ema5™). The Polyversum® WP preparation contains a fungus-like microorganism *Pythium oligandrum*. Moreover, in the autumn after leaf drop, the leaves were sprayed with a mixture of EmFarma Plus™ (20 l ha^−1^) and Ema5™ (2%) plus urea (1%) preparations.

**Table 1:** Combinations of foliar protective treatments during the hazelnut growing season in 2017 and 2018.

Combination of protection	Phenological phase (BBCH)								
	69^^^	71	75	78	80	82	85	88	90
Control	–	–	tiuram^1^	mankozeb^2^	tiuram	tiuram	–	–	–
Probiotics (EM) Polyversum^®^ WP	Bio 1^3^ + Bio 2^4^ *Pythium oligandrum*^5^	Bio 1 + Bio 2 *Pythium oligandrum*	tiuram tiuram	mankozeb mankozeb	tiuram tiuram	tiuram tiuram	Bio 1 + Bio 2 *Pythium oligandrum*	Bio 1 + Bio 2 *Pythium oligandrum*	Bio 1 + Bio 2 *Pythium oligandrum*

**Notes:**

1 Sadoplon 75 WP (tiura–75%), Synthos Agro Sp. z o.o., Poland, a dose of 3.4 kg ha^−1^.

2 Indofil 80 WP (mancozeb–80%), Indofil Industries Limited, Milan, the Italian Republic, a dose of 2.4 kg·ha^−1^.

3 EmFarma Plus™ (10^9^ colony-forming unit ml^−1^ mother cultures of living microorganisms SCD ProBio Plus®, organic sugar cane molasses, revitalised unchlorinated water, salt, a complex of minerals–0.2%), ProBiotics™ Poland, a dose of 10 l ha^−1^ in the BBCH phase of 69 and 71, 20 l ha^−1^ in the BBCH phase of 85, 88 and 90.

4 Ema5™ (10^9^ colony-forming unit ml^−1^ lactic acid bacteria, photosynthetic bacteria, fermenting fungi, yeasts, organic sugar cane molasses, wine vinegar, ethyl alcohol and revitalised unchlorinated water–0.2%), ProBiotics™ Polska, a dose of 10 l ha^−1^ in the BBCH phases 69 and 71, 20 l ha^−1^ in the BBCH phases of 85, 88 and 90.

5 Polyversum® WP, (10^6^ oospores of the fungus *Pythium oligandrum*-1 g in 1 litre of the preparation), Biopreparaty Sp. z o.o., the Czech Republic, a dose of 200 g ha^−1^.

^ Phenological phase: (BBCH) 69–end of flowering, 71–beginning of ovary growing, 75–beginning of fruit development, 78–85–fruit developed but mostly unripen, 88–90–fruit mostly fully ripe.

### Harvesting and storage of nuts

After harvest of hazelnuts from each treatments at the beginning of September 2017 and 2018, they were pre-cooled at 5 °C for 24 h and then stored in the experimental cold store at the Department of Horticulture, University of Warmia and Mazury in Olsztyn. The hazelnuts were stored for 3 months under the following conditions: normal atmosphere (NA, standard cold storage, at 0–1 °C, and humidity of 85–95%) and controlled atmosphere (CA, 3%O_2_:3%CO_2_, at 0–1 °C, and humidity of 85–95%). The experiment was set up in three replications in two consecutive years. In the first replicate there were 20 kg of nuts which were placed in airtight polyethylene (PE) containers with plexiglass (PMMA) lids with a capacity of 0.3 m^3^. Additionally 3 kg batches of the nuts per replication were placed in Xtend® MA/MH Packaging bags (MAP, Modified Atmosphere Packaging Bags, StePac, Israel) intended for stone fruit with an allowed weight of up to 5 kg, and stored under the NA conditions. The experiment was set up in three replications in two consecutive years. The Xtend® bags with nuts were stored in a normal atmosphere, at 0–1 °C, and humidity of 85–95%. The humidity in Xtend® bags during the storage of nuts ranged from 90–97%. The composition of the atmosphere (O_2_ and CO_2_) in Xtend^®^ bags was measured twice before they were opened (after 2 and 3 months of storage) (Table 2). The measurements were performed in triplicates using a gas analyser (WITT OXYBABY® M+O_2_/CO_2_) for controlling the quality of packaging intended for the storage of fruit in a modified atmosphere. Additionally, after being removed from storage, the chambers with the nuts in the husk were placed at room temperature (20 °C, and 40–60% relative humidity) for 7 days.

**Table 2:** The composition of the atmosphere (in %) in Xtend® bags (MAP) after 2- and 3-month storage of hazelnuts in the husk in 2017 and 2018.

Combination of protection	Period of storage											
	2017				2018				Means from years 2017–2018			
	2nd month		3rd month		2nd month		3rd month		2nd month		3rd month	
	CO_2_	O_2_	CO_2_	O_2_	CO_2_	O_2_	CO_2_	O_2_	CO_2_	O_2_	CO_2_	O_2_
Control	6.1	15.4	4.7	17.0	5.4	16.0	6.3	13.4	5.8	15.7	5.5	15.2
Probiotics (EM)	4.2	17.1	7.5	13.8	5.7	15.7	6.0	14.2	5.0	16.4	6.8	14.0
Polyversum® WP	5.5	16.5	7.1	14.2	6.0	15.3	6.7	13.3	5.8	15.9	6.9	13.8

### Assessment of nut morphological features

After 2 and 3 months of storage, the number and percentage of nuts in the husk and without husk were determined. The hazelnuts in the husk, and after separating the husk, were weighed, and after the hazelnuts were shelled, the weight of kernels was taken and the percentage of kernel was calculated. The dry matter of the kernels was determined by the oven-drying method at 105 °C according to the Polish Standard (1990). Determination of dry matter content by the gravimetric method and the moisture content of the kernels were then calculated. Additionally, prior to the storage of hazelnuts, the moisture content of the husks was determined, and it amounted to 78%.

### Health status of hazelnuts

After 2 and 3 months of nut storage, a visual inspection was carried out, and the hazelnuts were classified into two groups: the commercial yield (healthy nuts with no symptoms of any storage diseases) and the non-commercial yield (hazelnuts with symptoms of non-infectious and infectious diseases caused by fungi and bacteria displayed by symptoms on the husks and the nut shells). The visual inspection of the hazelnut quality was carried out on 6 kg samples in three replications. The obtained weights of the nuts in a particular classification group were converted into a percentage value.

Pathogenic fungi were isolated from diseased the hazelnuts and husks by plating pieces of infected tissues onto plates containing potato dextrose media (PDA, Merck, Poland). Microorganism were identified based on characteristic sporulation. The hazelnuts displaying symptoms of physiological diseases were characterized by visible features husks and shells appearing overrip, which mainly resulted in a change of the external brown-green external colour to dark brown.

### Bacterial and fungal colonization of hazelnuts

Portion of 10-g of randomly selected nuts with their husks were placed into a 250 cm^3^ flasks filled with 90 cm^3^ of sterile water. Fungi and bacteria were washed from the surface of nuts by shaking the flasks in a table shaker (Elpin Plus, Poland) for 30 min. Microorganism suspensions of 0.1 cm^3^ were plated onto 9 cm diameter Petri dish containing Martin medium (Martin, 1950) or enriched agar (EA). Fungi were identified based on the colony morphology and characteristic sporulation.

### Bacterial and fungal pathogenicity

Koch’s tests were conducted for eight pathogenic fungal isolates and nine bacterial isolates. Selected fungal isolates were repeatedly passaged on the PDA medium and bacterial isolates on the enriched agar (EA) in order obtain pure cultures. A drop of 0.1 cm^3^ of a fungal spore or bacterial cell suspensions of 10^6^–10^8^ spores/cell per ml was applied onto a cut of a leaf of the ‘Kataloński’ hazelnut kept in 9 cm diameter Petri dish moist tissue paper. Following inoculation, brown discoloured lesions were observed and recorded on the leaves after incubation for 7 days at 24 °C. The results were expressed as the percentage of infected leaf blade. Fragments were cut out from the infected leaf tissues and placed on the PDA medium after surface sterilization. Growing fungal colonies were identified based on spore morphology. The fragments of leaves inoculated with bacteria were ground in a mortar, and the obtained suspension was applied on Petri dishes which were filled with the EA medium.

### Molecular identification of bacteria

The DNA of bacterial isolates with varied virulence against the hazel nut leaves was isolated using a Bead-Beat Micro AX Gravity kit (A & A Biotechnology, Gdańsk, Poland) according to the manufacturer’s procedure. The quantity and quality of the isolated DNA were determined by measuring absorbance at a wavelength of 260 nm and 280 nm (NanoMaester Gen, Poland). Each 50 μL PCR reaction mixture consisted of genomic DNA (50 ng), 23 μL 2xPCR Tag/NovaRED (Blirt, Poland), 1 μL forward primer (27F 5′-AGAGTTTGATCCTGGCTCAG-3′), and 1 μL reverse primer (1492R 5′-GGTTACCTTGTTACGACTT-3′) and 25 μL water. PCR was performed in a thermocycler machine (Eppendorf, Poland) with the following cycling conditions: an initial denaturation at 95 °C for 6 min, 40 cycles of denaturation at 95 °C for 30 s, annealing at 50 °C for 1 min, and extension at 72 °C for 1 min. A final extension was performed at 72 °C for 10 min. PCR products were analysed using 1.5% agarose gel electrophoresis. The nucleotide sequences were compared with reference retrieved in BLAST searches of GenBank (Wachowska et al., 2013).

### Data analysis

The results obtained in the experiment were statistically analysed by a one- and two-factor variance analysis (ANOVA). Regards the morphological features, separation of means was carried out with Tukey’s test at *P* < 0.05%; the analysed data included the 2 years, 2017 and 2018. The data on fungal, bacterial and yeast counts, and their pathogenicity were analysed based on the separation of means with the Tukey’s test at *P* < 0.01%.

## Results

### Analysis of the gas composition in Xtend® bags

The measurement of the gas composition in Xtend® bags (MAP) after 2 and 3 months of storage of the hazelnuts protected before harvesting with probiotics and the preparation Polyversum® WP showed an increase in CO_2_ content and a decrease in O_2_ content (Table 2). In the bags with nuts unprotected before harvesting, the average CO_2_ and O_2_ contents after 3 months of storage remained at a similar level.

### Assessment of the morphological features of the nuts after storage

Immediately after removing the nuts from the storeroom, the average weight of hazelnuts in the husk and without husk, the kernel weight, and the average moisture content of the kernels of nuts stored under the CA conditions and in Xtend® bags (MAP) were all significantly greater than those under the NA conditions after both 2 and 3 months of storage (Table 3). The application of probiotics before harvesting the nuts significantly contributed to improved maintenance of the hazelnut kernel weight after 2 and 3 months of storage and a higher percentage of kernel after 3 months of storage compared to the control. After 2 months of storage, the percentage of kernel originating from trees protected before harvesting with the preparation Polyversum® WP was significantly greater than that of the hazelnuts obtained from unprotected trees. After 3 months of storage in the CA, the weight and the percentage of kernel were significantly higher in combination with the protection using *P. oligandrum* than in the untreated control.

**Table 3:** Morphological features of hazelnuts after harvesting and after 2 and 3 months of storage in 2017 and 2018.

Combination of protection	Period and condition of storage after:								
	2 months				3 months				Means
	NA	CA	MAP	Means	NA	CA	MAP	Means	
Weight of hazelnuts in the husk (g)									
Control Probiotics (EM) Polyversum® WP	5.5^cd^ 5.5^cd^ 5.1^d^	6.3^a^ 6.0^abc^ 6.2^ab^	5.7^bc^ 6.5^a^ 6.1^ab^	5.8^**a**^ 6.0^**a**^ 5.8^**a**^	4.9^c^ 5.2^c^ 4.8^c^	6.0^b^ 6.1^ab^ 6.5 ^a^	6.5^a^ 6.1^ab^ 6.0^b^	5.8^**a**^ 5.8^**a**^ 5.7^**a**^	5.8^*a*^ 5.9^*a*^ 5.8^*a*^
Means (condition)	5.4^B^	6.2^A^	6.1^A^		4.9^C^	6.2^A^	6.2^A^		
Means (period)		5.9^A_^				5.8^A_^			
Weight of hazelnuts without husk (g)									
Control	4.0^cd^	4.3^abc^	3.8^d^	4.0^**b**^	3.8^ef^	4.1^cde^	4.5^a^	4.1^ab^	4.0^*b*^
Probiotics (EM)	4.1^bcd^	4.2^bc^	4.5^a^	4.3^**a**^	4.0^def^	4.2^bcd^	4.3^abc^	4.2^ab^	4.2^*a*^
Polyversum® WP	3.8^d^	4.3^abc^	4.3^abc^	4.1^**ab**^	3.7^f^	4.5^a^	4.2^bcd^	4.1^ab^	4.1^*ab*^
Means (condition)	4.0^B^	4.3^A^	4.2^A^		3.8^B^	4.3^A^	4.3^A^		
Means (period)		4.2^A_^				4.1^A_^			
Weight of hazelnut kernels (g)									
Control	1.8^bc^	1.9^ab^	1.6^c^	1.8^**b**^	1.7^cd^	1.8^bc^	1.9^bcd^	1.8^**b**^	1.7^*b*^
Probiotics (EM)	1.8^bc^	1.9^ab^	2.0^a^	1.9^**a**^	1.8^bc^	1.9^bcd^	2.0^ab^	1.9^**a**^	1.9^*a*^
Polyversum® WP	1.6^c^	2.0^a^	2.0^a^	1.9^**a**^	1.5^d^	2.1^a^	1.9^bcd^	1.8^**b**^	1.8^*ab*^
Means (condition)	1.7^B^	1.9^A^	1.9^A^		1.6^B^	1.9^A^	1.9^A^		
Means (period)		1.8^A_^				1.8^A_^			
Percentage of kernel (%)									
Control	43.6^bc^	45.0^abc^	40.3^d^	43.0^**bc**^	43.7^ab^	42.9^ab^	41.8^bc^	42.8^**c**^	42.9^*b*^
Probiotics (EM)	43.3^bcd^	46.0^ab^	44.4^bc^	44.6^**abc**^	44.6^ab^	44.1^ab^	45.6^a^	44.7^**ab**^	44.6^*a*^
Polyversum® WP	42.1^cd^	45.2^abc^	47.5^a^	45.0^**a**^	40.8^c^	45.8^a^	43.9^ab^	43.5^**abc**^	44.2^*a*^
Means (condition)	43.0^B^	45.4^A^	44.1^AB^		43.0^A^	44.2^AB^	43.8^AB^		
Means (period)		44.2^A_^				43.7^A_^			
Moisture content of kernel (%)									
Control Probiotics (EM) Polyversum® WP	40.2^bc^ 38.3^c^ 48.0^ab^	50.5^a^ 48.6^a^	50.2^a^	47.0^**a**^	44.7^a^	51.1^a^	45.0^a^	46.9^**a**^	47.0^*ab*^
			47.2^ab^	46.9^**a**^	40.1^a^	49.5^a^	51.4^a^	47.0^**a**^	45.8^*b*^
		53.5^a^	48.2^ab^	49.9^**a**^	43.5^a^	51.3^a^	52.2^a^	49.0^**a**^	49.4^*a*^
Means (condition)	42.2^B^	50.9^A^	48.5^A^		42.8^B^	50.6^A^	49.5^A^		
Means (period)		47.2^A_^				47.6^A_^			

**Note:**

The mean values within individual period of storage marked with the same indices do not differ significantly at *P* < 0.05, HSD Tukey test; a, b, c… homogenous groups, interaction combination of protect and condition of storage, **a, b, c**… homogenous groups, main effect of combination of protect for each storage period saparately, *a,b,c*… homogenous groups, main effect of combination of protect for all storage periods, A, B, C… homogenous groups, main effect of condition for each storage periods saparately, A, B, C… homogenous groups, main effect of storage period.

The assessment of morphological features of the hazelnuts stored for 7 days at room temperature after removing them from the cold storage under CA conditions and Xtend® bags after 3 months demonstrated that the analysed morphological parameters were retained at a level higher than that for the control (Table 4). The hazelnuts originating from trees protected with the preparation Polyversum® WP stored for 3 months in Xtend® bags had a greater weight in the husk and without husk and the weight of the kernel, and a higher percentage of kernel than the nuts unprotected before harvesting and stored under the NA conditions.

**Table 4:** Morphological features of hazelnuts stored for 7 days at room temperature after being stored in 2017 and 2018.

Combinations of protection	Period and condition of storage after:								
	2 months				3 months				Means
	NA	CA	MAP	Means	NA	CA	MAP	Means	
Weight of hazelnuts in the husk (g)									
Control	3.3^c^	4.2^a^	3.4^c^	3.6^**b**^	3.3^e^	4.1^a^	3.6^cd^	3.7^**ab**^	3.6^*b*^
Probiotics (EM)	3.8^bc^	3.9^b^	3.9^b^	3.8^**a**^	3.4^de^	3.7^cd^	3.8^bc^	3.6^**b**^	3.7^*ab*^
Polyversum® WP	3.7^bc^	4.0^ab^	3.5^cd^	3.7^**ab**^	3.6^cd^	4.0^ab^	4.1^a^	3.9^**a**^	3.8^*a*^
Means (condition)	3.6^C^	4.0^A^	3.6^C^		3.4^D^	3.9^AB^	3.8^B^		
Means (period)		3.7^A_^				3.7^A_^			
Weight of hazelnuts without husk (g)									
Control	2.9^d^	3.7^a^	2.9^d^	3.2^**b**^	2.9^e^	3.6^a^	3.1^de^	3.2^**b**^	3.1^*b*^
Probiotics (EM)	3.3^bc^	3.4^b^	3.4^b^	3.4^**a**^	3.0^de^	3.3^bc^	3.3^bc^	3.2^**b**^	3.2^*ab*^
Polyversum® WP	3.2^bc^	3.5^ab^	3.1^cd^	3.3^**ab**^	3.2^cd^	3.5^ab^	3.5^ab^	3.4^**a**^	3.3^*a*^
Means (condition)	3.1^DE^	3.5^A^	3.2^CD^		3.0^E^	3.4^AB^	3.3^BC^		
Means (period)		3.3^A_^				3.2^A_^			
Weight of hazelnut kernels (g)									
Control	1.1^f^	1.5^a^	1.1^f^	1.2^**b**^	1.1^e^	1.5^a^	1.1^e^	1.2^**b**^	1.2^*b*^
Probiotics (EM)	1.3^bcd^	1.3^bcd^	1.4^ab^	1.3^**a**^	1.1^e^	1.3^bc^	1.2^cde^	1.2^**b**^	1.3^*a*^
Polyversum® WP	1.2^de^	1.4^ab^	1.2^de^	1.3^**a**^	1.2^cde^	1.3^bc^	1.4^ab^	1.3^**a**^	1.3^*a*^
Means (condition)	1.2^BC^	1.4^A^	1.3^B^		1.0^C^	1.4^A^	1.3^B^		
Means (period)		1.3^A_^				1.2^B_^			
Percentage of kernel (%)									
Control	36.8^d^	40.8^ab^	36.4^d^	38.0^**ab**^	37.5^bc^	40.7^a^	35.8^c^	38.0^**ab**^	38.0^*a*^
Probiotics (EM)	38.0^bcd^	38.8^a-d^	41.3^a^	39.4^**a**^	36.7^bc^	38.1^abc^	36.6^bc^	37.1^**b**^	38.3^*a*^
Polyversum® WP	37.7^cd^	40.6^abc^	40.0^abc^	39.4^**a**^	38.3^abc^	36.7^bc^	39.1^ab^	38.0^**ab**^	38.7^*a*^
Means (condition)	37.5^C^	40.0^A^	39.2^AB^		37.5^C^	38.5^BC^	37.1^C^		
Means (period)		38.9^A_^				37.7^B_^			
Moisture content of kernel (%)									
Control	17.5^a^	27.2^a^	21.9^a^	22.2^**a**^	13.7^b^	26.4^a^	15.3^b^	18.5^**a**^	20.4^*a*^
Probiotics (EM)	20.1^a^	25.0^a^	23.0^a^	22.7^**a**^	16.6^b^	21.8^ab^	19.1^ab^	19.2^**a**^	20.9^*a*^
Polyversum® WP	21.1^a^	26.5^a^	18.4^a^	22.0^**a**^	15.8^b^	26.3^a^	21.3^ab^	21.1^**a**^	21.6^*a*^
Means (condition)	19.6^BC^	26.2^A^	21.1^AB^		14.4^C^	24.8^A^	18.6^BC^		
Means (period)		22.3^A_^				19.6^B_^			

**Note:**

Explanation, see Table 3.

**Figure 1:**
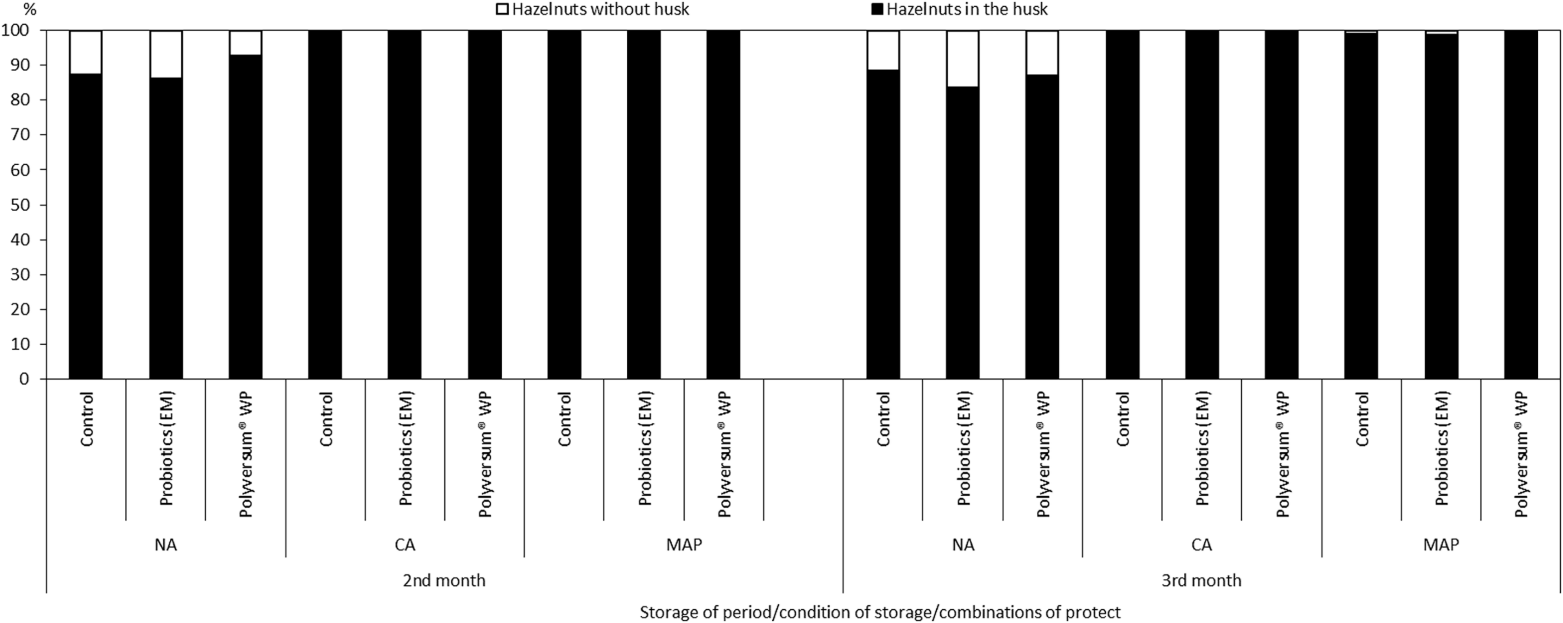
The effect of biopreparations on the percentage of hazelnuts in the husk and without husk, depending on the storage conditions and period in the years 2017–2018. NA, normal atmosphere; CA, controlled atmosphere; MAP-Xtend®, packaging bags (0–1 °C, 85–95% RH).

The nuts stored under the NA conditions exhibited the highest degree of husk separation from the shell (Fig. 1). This phenomenon was due to rapid overriping of hazelnuts under these conditions, which was confirmed by an analysis of the composition of the atmosphere during the storage of nuts in Xtend® bags (Table 1).

### Health status of hazelnuts

**Figure 2:**
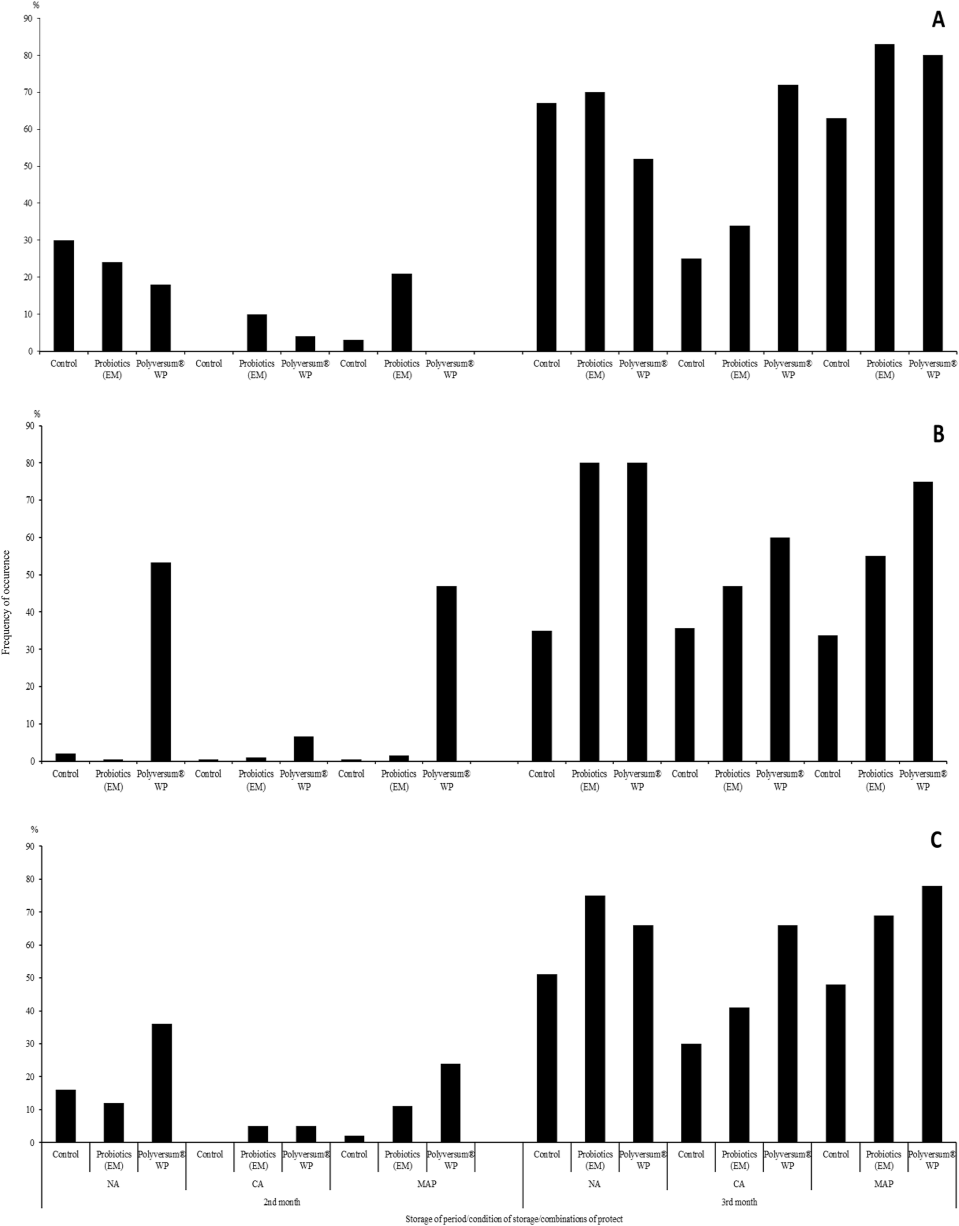
Effects of biopreparations on the frequency of occurrence of hazelnuts in the husk displaying symptoms of infectious and physiological diseases, depending on the storage conditions and period in the years 2017–2018 NA, normal atmosphere; CA, controlled. NA, normal atmosphere; CA, controlled atmosphere; MAP-Xtend®, packaging bags (0–1 °C, 85–95% RH). (A) Means from year 2017, (B) means from year 2018, (C) means from years 2017–2018.

After 3 months of storage, numerous symptoms of infectious and non-infectious diseases were observed on the husks and hazelnut shells. The incidence of the nuts with disease ranged from 83–92% for the nuts stored under the NA conditions and from 99–100% for those stored under the CA conditions and in Xtend® bags (MAP) (Fig. 2). Samples stored under the regular cold storage conditions (NA) had the highest percentage of nuts with diseases (Fig. 2). However, hazelnuts stored under the CA conditions displayed the lowest incidence of diseases. The application of biopreparations during the growing period resulted to a higher percentage of nuts displaying symptoms of infectious and non-infectious diseases than the untreated control. The application of Polyversum® WP, resulted in the greatest production of nuts classified as non-commercial nuts.

### Hazelnut and husk microbiome

**Figure 3:**
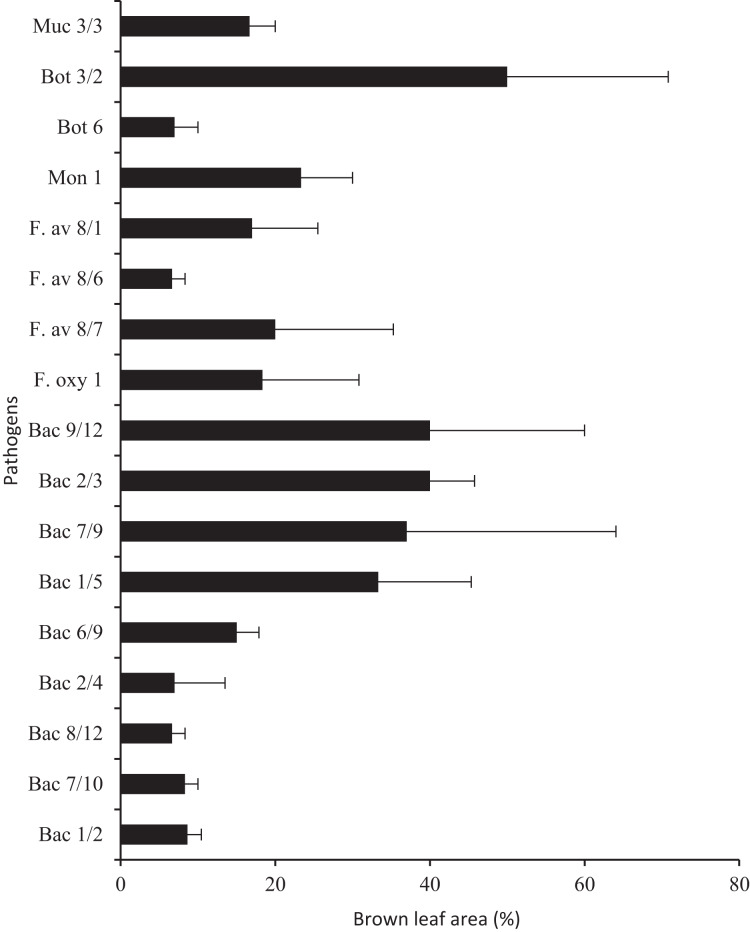
Pathogenicity of selected fungal and bacterial isolates tested on hazelnut leaves (the percentage of leaf blades displaying symptoms of infection). Muc, *Mucor* sp.; Bot, *Botrytis* sp.; Mon, *Monilia* sp.; F. av, *Fusarium avenaceum;* F. oxy, *Fusarium oxysporum*,; Bac, bacteria.

Protective treatments performed using probiotics and the Polyversum® WP preparation showed no reduction of nuts showing symptoms of physiological and infectious diseases (Fig. 2C). The following species and genera of pathogenic fungi were isolated from the nuts and husks displaying symptoms of infectious diseases: *Fusarium avenaceum*, *F. oxysporum*, *Botrytis* spp., *Mucor* spp., *Monilia* spp. (Table 5). In general, the storage and protection methods applied had no significant effect on the mean pathogen count. Only after the application of the preparation Polyversum® WP under the NA conditions there was no presence of pathogenic fungi on nuts. The Koch postulatest test revealed that all of the identified pathogens to cause browning of the hazel nut leaf tissue (Fig. 3). The pathogen *Botrytis* sp. Bot 3/2 stood out due to the highest pathogenicity. After storing the hazelnuts for 3 months, a layer of dark-coloured hyphae of fungi of the *Alternaria* and *Cladosporium* genera was observed on the husk. In general, the analysed factors had no significant effect on incidence of *Alternaria* and *Cladosporium* genera (Table 5). Only the husks of nuts protected using Polyversum® WP in in Xtend® bags (MAP) showed significant higher counts of *Cladosporium* spp. than those in the untreated control husks. In the control, on the hazelnuts stored under the NA conditions, a considerable reduction in the count of *Penicillium spp*. was noted after the application of probiotics and Polyversum® WP. The yeast count significantly increased compared to the control on the nuts stored in Xtend® bags (MAP) and under the NA conditions in combination with the protection using Polyversum® WP.

**Table 5:** Counts of fungi, bacteria and yeasts colonising the surface of the husk after 3 months of storage, depending on the storage conditions and hazelnut protection methods in 2018.

Condition of storage	Combinations of protection	Composition of atmosphere		Pathogens*	*Alternaria* spp.	*Acremonium* spp.	*Cladosporium* spp.	*Penicillium* spp.	Bacteria	Yeast
		CO_2_	O_2_	Number of colonies × 10^2^ per 1 g of husk					Number of colonies × 10^4^ per 1 g of husk	
NA	Control	0	21.0	1.67^ab^	22.33^ab^	0.33^b^	0.00^c^	281.33^a^	7.68^f^	6.20^cd^
	Probiotics (EM)	0	21.0	8.67^a^	19.67^ab^	0.00^b^	21.66^bc^	5.66^b^	53.52^bc^	34.24^ab^
	Polyversum® WP	0	21.0	0.00^c^	17.00^ab^	0.33^b^	41.33^bc^	1.00^b^	40.35^d^	18.13^bcd^
CA	Control	3.0	3.0	1.33^ab^	6.00^b^	20.66^a^	86.66^bc^	1.66^b^	32.69^de^	1.96^d^
	Probiotics (EM)	3.0	3.0	1,67^ab^	20.00^ab^	3.66^b^	97.00^bc^	5.33^b^	28.69^de^	8.32^cd^
	Polyversum® WP	3.0	3.0	6.00^ab^	60.00^a^	1.66^b^	102.00^bc^	1.33^b^	72.11^ab^	26.19^abc^
MAP	Control	5.5	15.2	4.00^ab^	42.00^ab^	0.66^b^	124.00^b^	1.33^b^	17.23^ef^	11.78^cd^
	Probiotics (EM)	6.8	14.0	4.33^ab^	24.67^ab^	27.33^a^	26.66^bc^	2.66^b^	73.16^a^	46.93^a^
	Polyversum® WP	6.9	13.8	2.33^ab^	19.33^ab^	9.66^b^	298.66^a^	2.33^b^	39.89^cd^	21.92^bcd^
Means (condition)		NA		3.00	19.67	0.22^Y^	21.00^Y^	96.00^X^	33.84^X^	19.52^XY^
		CA		3.44	28.67	8.67^X^	95.22^Y^	2.78^Y^	44.49^Y^	12.15^Y^
		MAP		3.56	28.67	12.56^X^	149.78^X^	2.11^Y^	43.43^Y^	26.88^X^
Means (protection)		Control		2.33^B^	23.44	7.22^AB^	70.22^B^	94.78^A^	19.20^B^	6.64^B^
		Probiotics (EM)		4.89^A^	21.44	10.33^A^	48.44^B^	4.56^B^	51.79^A^	29.83^A^
		Polyversum® WP		2.78^B^	32.11	3.89^B^	147.33^A^	1.56^B^	50.78^A^	22.08^A^

**Notes:**

NA, normal atmosphere; CA, controlled atmosphere; MAP-Xtend®, packaging bags (0–1 °C, 85–95% RH).

The mean values marked with the same letters do not differ significantly at *P* < 0.05, HSD Tukey test; *- *Botrytis* sp., *Mucor* spp., *Fusarium* spp., *Monilia* sp.

A very large bacterial community was washed from the hazelnuts; generally, the count of bacteria increased significantly after the application of probiotics and Polyversum® WP and on the nuts stored in Xtend® bags (MAP) and under the CA conditions compared to unprotected nuts before harvesting and after storing under the NA conditions (Table 5). Five selected strains (Bac 9/12, 2/3, 1/5, 6/9 and 7/9) formed mucous, yellow, round, convex, smooth, and shiny colonies. The representative strain Bac 9/12 was identified based on the analysis of the ITS region sequence as *Pantoea agglomerans*. Five strains, (Bac 2/4, 8/12, 7/10 and 1/2) formed small, light-yellow colonies and a representative strain Bac 1/2 was classified as the *Arthrobacter luteolus*. After the application of *P. agglomerans* suspension on the leaves, brown lesions developed covering 15–40% of the leaf surface, after application of *A. luteolus* brown lesions covering and 7–9% of the leaf surface developed (Fig. 3).

## Discussion

Modern consumer market follows products that have high health-promoting potencial. Hazel grown to be a fresh nut that may be stored for 2 months without decline of health has an advantage in cold storage if enclosed in Xtend® bags perfectly and fits into such trend. Growing up hazel to be used as a fresh nut we obtain not only higher crop and lengthened nuts consumption period during the year, but also we provided market with nuts of higher gustatory advantage, higher polyphenol content and some fatty acids as well as macronutrients (Markuszewski, 2020). The current study showed that unripe hazelnuts stored in the husk under the controlled atmosphere conditions encloded in Xtend® bags (MAP) were exhibited greater commercial value both immediately after removal from the cold storare and after keeping them at room temperature 7 days after removing them from cold storage. This was consistent with previous opinions on the higher quality of hazelnuts stored in an atmosphere with lower O_2_ levels and higher CO_2_ levels (Keme et al., 1980; Moscetti et al., 2012; Ghirardello et al., 2013; Guiné, Almeida & Correia, 2015; Markuszewski & Kopytowski, 2015).

Hazelnuts treated with the preparation Polyversum® WP before harvest and stored had a greater weight without husk, a higher percentage of kernel and a higher moisture content of the kernel than kernels of control. In addition, the hazelnuts sprayed with Polyversum® WP during the storage in Xtend® bags (MAP) for 3 months exhibited a further increase in respiration processes. The obtained results confirm the stimulating effect of the preparation on unripe hazelnuts in the husk during storage. According to the manufacturer, (Biopreparaty Ltd., the Czech Republic), one of the effects of Polyversum® WP activity towards plants is the production of substances stimulating their growth and, indirectly, an increase in the phosphorus and micronutrient uptake by plants, which is reflected in an increased of plant growth.

In the current experiment, the hazelnuts were characterised by a high moisture content not only in the kernels (50%) but primarily in the husks (78%). At the same time, the storage of unripe hazelnuts for 3 months under 85–97% humidity, particularly in Xtend® bags (MAP), contributed to the development of infectious and non-infectious diseases, occurring mainly on the husks, resulting in the loss of commercial value. According to Şimşek, Arici & Demir (2002), Campbell, Molyneux & Schatzki (2003) and Navarro (2006), air humidity exceeding 70% during long-term storage promotes the infection of hazelnuts by fungal pathogens. In the present study, the higher percentage of nuts displaying symptoms of diseases, particularly under the NA conditions but also in Xtend® bags (MAP), was mainly related to higher O_2_ levels during the storage. Therefore, a low oxygen content and the presence of carbon dioxide during the storage of hazelnuts under the CA conditions can effectively inhibit their respiration (Keme et al., 1980; San Martin et al., 2001; Ghirardello et al., 2013) while limiting the development of fungal and physiological diseases (Leuty et al., 2012). The quality of hazelnuts stored under conditions of excessively high or low humidity may gradually deteriorate (Moscetti et al., 2012; Markuszewski et al., 2017).

During the growing season in Poland, hazelnuts are infected by several fungal pathogens such as *Monilia* spp., *Botrytis cinerea*, *Phyllactinia corylea*, *Cryptosporiopsis coryli* and *Cladosporium avellanum* (Machowicz-Stefaniak & Zalewska, 2000). In 2007, symptoms of bacterial blight induced by *Xanthomonas arboricola* pv. *corylina* (Xac) were noted as well (Pulawska et al., 2010). After 3 months of storage, the occurrence of pathogens of the *Alternaria*, *Botrytis, Monilia, Mucor* and *Fusarium* species, whose counts were generally not reduced by either the storage methods or the protective treatments applied before harvesting, found on the hazelnuts and their husks. Only under the NA conditions in combination with the preparation Polyversum® WP the above-mentioned pathogens were not found. Previous studies indicated that the application of Polyversum® WP during the plant growing period exhibited antagonistic activity against pathogenic fungi, such as *Alternaria, Botrytis, Fusarium* and *Sclerotinia* genera and others (Rekanovic et al., 2007; Wang, Lou & Xu, 2011).

In this study, the very high count of fungi of the *Alternaria* and *Cladosporium* genera which formed a dark-coloured layer on the hazelnut husks was puzzling. The increase in the count of *Cladosporium* spp. and the simultaneous reduction in the number of *Penicillium* spp. under the influence of the analysed factors, particularly on the nuts stored in Xtend® bags (MAP), was an undwaired phenomenon. These species, previously identified on hazelnuts by Abdel-Hafez & Saber (1993), were only considered as minor contributors to post-harvest disease. Currently, however, numerous studies indicate that *Cladosporium* spp. can cause a number fruit diseases during storage period, such as brown spot of table grape (*Vitis vinifera* L.) (Swett, Bourret & Gubler, 2017) and *Cladosporium* fruit rot of red raspberry (*Rubus idaeus* L.) (Swett et al., 2019). However, the same authors suggested that in order for fruits to be infected, fruit micro-wounds caused by insects were necessary. *Cladosporium* spp. though are not usually considered to be the cause of economically significant decreases in fruit yields (Ellis et al., 1991) and thus there is very limited information on management to limit these diseases. Studies by Ellis et al. (1991) and Swett, Bourret & Gubler (2017) indicated that fungicide treatments are not effective in limiting fruit rot caused by *Cladosporium* spp.

*Alernaria* fungi presented in this research emerged on nuts stored for 3 months in high incidence and the way of storage had basically no influence on decay. In research conducted in China *A. alternata* species were related to an internal form of hazelnut apical necrosis (Cheng et al., 2017), and in Chile that pathogen caused grey necrosis of hazelnut (Duran et al., 2020). Species of *Alternaria* can produce up to 30 different metabolites, whence alternariol (AOH), alternariol monomethyl ether (AME), alteuene (ALT), tenuazonic acid (TeA), tentoxin (TEN), and altertoxins I, II and III are described as metabolites with toxic act on systems (Lee, Patriarca & Magan, 2015). No report about detecting mycotoxins such as aflatoxin produced by *Aspergillus flavus* until now when nuts stored in Turkey, were found to be contaminated by alfatoxins (Ozay et al., 2008). Certainly the presence of *Alternaria* fungi on hazelnuts is highly undesired.

This research of nuts stored for 3 months showed only occasionally *Fusarium* fungi. Particular danger for quality of the product may be posed by isolated *F. avenceum* from infected hazelnuts. This pathogen produces over a dozen mycotoxins, including monoliforomin, enniatin and beauvericin (Christ, Märländer & Varrelmann, 2011). *Fusarium* pathogens are rarely described as culprits of nut diseases. In Italy *F. lateritium* caused yellow-to-orange sporodochium on branches of walnuts (Belisario, Maccaroni & Coramusi, 2005). No *Fusarium* mycotoxins have been detected in Poland, however aflatoxins have been detected. In European Union, the maximum content (tolerance) of total aflatoxins in nuts intended for consumption is 10 µg/kg (Hadi & Kashefi, 2012).

In this study, the authors found the occurrence of a large number of yeast and bacterial colonies on the hazelnuts and their husks; a significantly greater number of these microorganisms were isolated from the hazelnuts treated with biological preparations. Yeasts are commonly used to limit the development of fruit diseases. They compete for space and nutrients with pathogens while limiting the development of the pathogen microorganism. In particular, the combination of these fungi with UV-C has a positive effect in limiting disease symptoms on freshly cut pineapples (Ou et al., 2016), melons (Huang et al., 2015), peaches (Xu & Du, 2012) and kiwi fruits (Tang et al., 2015). On the other hand, the yeast *Nematospora coryli* (*Eremothecium coryli*) was isolated from hazelnuts (*Corylus avellana*), and this species was found to be responsible for causing kernel spot (Bobev et al., 2018).

The isolated bacterial strains in this study from unripe hazelnuts and their husks belong to the *P. agglomerans* species. In the Koch postulates tests 7 days after incubating the hazelnut leaves in a humid chamber, necrosis of the tissue was observed. The pathogenic strains were classified as *P. agglomerans* based on the analysis of the 16S rDNA sequence. The results of this study suggest that during the storage of hazelnuts in the husk under high humidity conditions, these bacteria may cause necroses of the husks. Medarno & Bell (2007) found that isolates of this species originating from cotton bolls any disease symptoms displayed diseases symptoms 2 weeks only after infecting the cotton bolls with abacterial suspension. These authors concluded that bacteria of this species can infect cotton seed bolls under field conditions following damage by insects feeding. Moreover, Lu, Jia & Gao (2015) described for the first time a strain belonging to the *P. agglomerans* species which caused bacterial leaf blight of *Vigna angularis* in the Jilin Province in China.

## Conclusions

Extending the period of selling fresh hazelnut in the husk is very desirable for its producers and consumers. This research showed that the 2 months’ storage of hazelnuts in a controlled atmosphere and their enclosure in Xtend® bags (MAP) prevented a reduction in the weight of hazelnuts in the husk, without husk, and of the kernel and prevented the nut separation from the husk. In general, the application of biopreparations in the protection of the hazelnut had a positive effect on the kernel weight and the percentage of kernel.

This research also is the first report in Poland in which *Cladosporium*, *Alternaria* and *Fusarium* spp. Caused disease of hazelnuts after 3 months of storage, resulting in the loss of the commercial value. To solve the problems associated with storage of nuts in the husk there is a need for additional research in improving storage conditions and expanding the late autumn season for the hazelnuts industry. Such efforts may be supported by, at least partly, better understanding of the impact of storage on pathogens and quality of nuts.

## Supplemental Information

10.7717/peerj.12760/supp-1 Supplemental Information 1 Raw data for Table 5.

10.7717/peerj.12760/supp-2 Supplemental Information 2 Raw data.

10.7717/peerj.12760/supp-3 Supplemental Information 3 Raw data for Pathogenicity of selected fungal and bacterial isolates tested on hazelnut leaves.

## Additional Information and Declarations

### Data Availability

The following information was supplied regarding data availability: The raw data is available in the Supplemental Files.
